# Enhancing CAR T‐cell therapy manufacturing efficiency through semi‐automated bioprocessing

**DOI:** 10.1002/cti2.70025

**Published:** 2025-06-02

**Authors:** Jason Isaacson, Prajakta Bhanap, Nicholas Putnam, Jasmine Padilla, Nujhat Fatima, Max Dotson, Danny Hayoun, Moloud Ahmadi, Gertrude Nonterah, Yongchang Ji

**Affiliations:** ^1^ Thermo Fisher Scientific Carlsbad CA USA

**Keywords:** automation, CAR T‐cell manufacturing, CAR T cells, cell therapy

## Abstract

**Objectives:**

Chimeric antigen receptor (CAR) T‐cell therapies have revolutionised the treatment of blood‐based malignancies. The use of manual CAR T‐cell manufacturing methods is one of the challenges that contributes to these delays. As CAR T therapy emerges as a potential first‐ or second‐line treatment option for these cancers, the demand for these therapies continues to rise. However, challenges persist in ensuring that the patients who need these therapies receive them in a timely manner. Automated CAR T‐cell manufacturing methods that use software to control the equipment used in the process can help overcome the roadblocks associated with manual manufacturing, ultimately enabling a reduction in variability, increased efficiency, improved product quality and better data management. This paper aims to present an end‐to‐end semi‐automated methodology for manufacturing CAR T cells using the Cell Therapy Systems (CTS™) Cellmation software – an off‐the‐shelf software solution – to control physically connected modular cell therapy instruments that eliminates the roadblocks associated with manual manufacturing.

**Methods:**

T cells from healthy donors were isolated and processed into CAR T cells using a semi‐automated, connected, multi‐instrument setup that leveraged electroporation and a CRISPR/Cas system for delivering the CD19‐CAR construct to the T cells. Flow cytometry was used to assess cell type composition, cell viability and expression of T‐cell activation markers throughout the process. We also measured exhaustion marker expression on T cells, T‐cell receptor (TCR) knock‐out, CAR knock‐in and cytotoxic activity against NALM6 cells.

**Results:**

The results demonstrated the successful generation of functional CAR T cells using a semi‐automated instrument workflow. The results were similar to the results from CAR T cells manufactured using non‐automated processes; however, the successful connection and control of the instruments using automated software present an exciting opportunity for process developers and manufacturers who want to reduce manual touchpoints in their cell therapy manufacturing process.

**Conclusion:**

The method that we describe in this paper could be beneficial to process development and manufacturing teams that might require flexibility in their CAR T cell manufacturing workflow and want to take advantage of modular systems that can be automated using the Cellmation software to reduce the problems associated with manual handling.

## Graphical abstract

We present an end‐to‐end methodology for semi‐automating the cell therapy manufacturing workflow using CTS Cellmation software – an off‐the‐shelf software solution – to control physically connected modular cell therapy instruments.

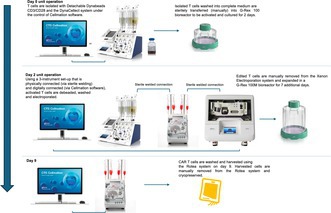

## Introduction

Chimeric antigen receptor (CAR) T cells are a groundbreaking and lifesaving treatment for patients with haematological malignancies. While CAR T therapy was once a ‘last line of defence’ treatment for these cancers, it is emerging as a treatment that could be leveraged earlier on in cancer.^1^, ^2^ With these remarkable breakthroughs come an increase in patients who are eligible to receive CAR T‐cell therapies, and this upward trend is likely to continue.

However, challenges remain in ensuring these therapies get to the patients who need them the most. While some of these roadblocks can be related to supply chain and logistics problems, it is often problems encountered in the manufacturing process that can lead to these delays.

Using multiple modular instruments in the cell therapy manufacturing workflow provides manufacturers with flexibility and increased scalability compared with all‐in‐one manufacturing systems. Without an automation solution that controls the overall process, however, having multiple instruments could introduce several operator touchpoints which can increase the likelihood of contamination and error. This can introduce unnecessary delays in the cell therapy production process.

Furthermore, the absence of automation software that controls instruments in a cell therapy workflow can hamper traceability data and lead to the violation of cGMP guidelines.

Simplifying and streamlining the cell therapy manufacturing process is necessary to curb these problems, and it can be achieved through automation. Automated bioprocesses help to do the following:

1. Reduce error and variability: Variability can have a profound effect on product quality and function. Automation helps ensure that each batch of CAR T cells is produced at the same high standard. For cell therapies in the pre‐approval stage, any change, regardless of how minor, can result in an alteration of product quality, affecting the safety and efficacy of the therapy, and prolong time to approval.
2. Eliminate human error: Different operators on different shifts, for instance, can inadvertently introduce errors and compromise manual cell therapy manufacturing processes.
3. Increase efficiency and scalability: Automated systems can handle and process larger volumes of cells. This can help enable manufacturers to meet the increasing demand for cell therapies.
4. Enable better process control, monitoring and data management: The data collected during the cell therapy manufacturing process is easily traceable, is reproducible and can be securely stored using automation software.

**Figure 1:**
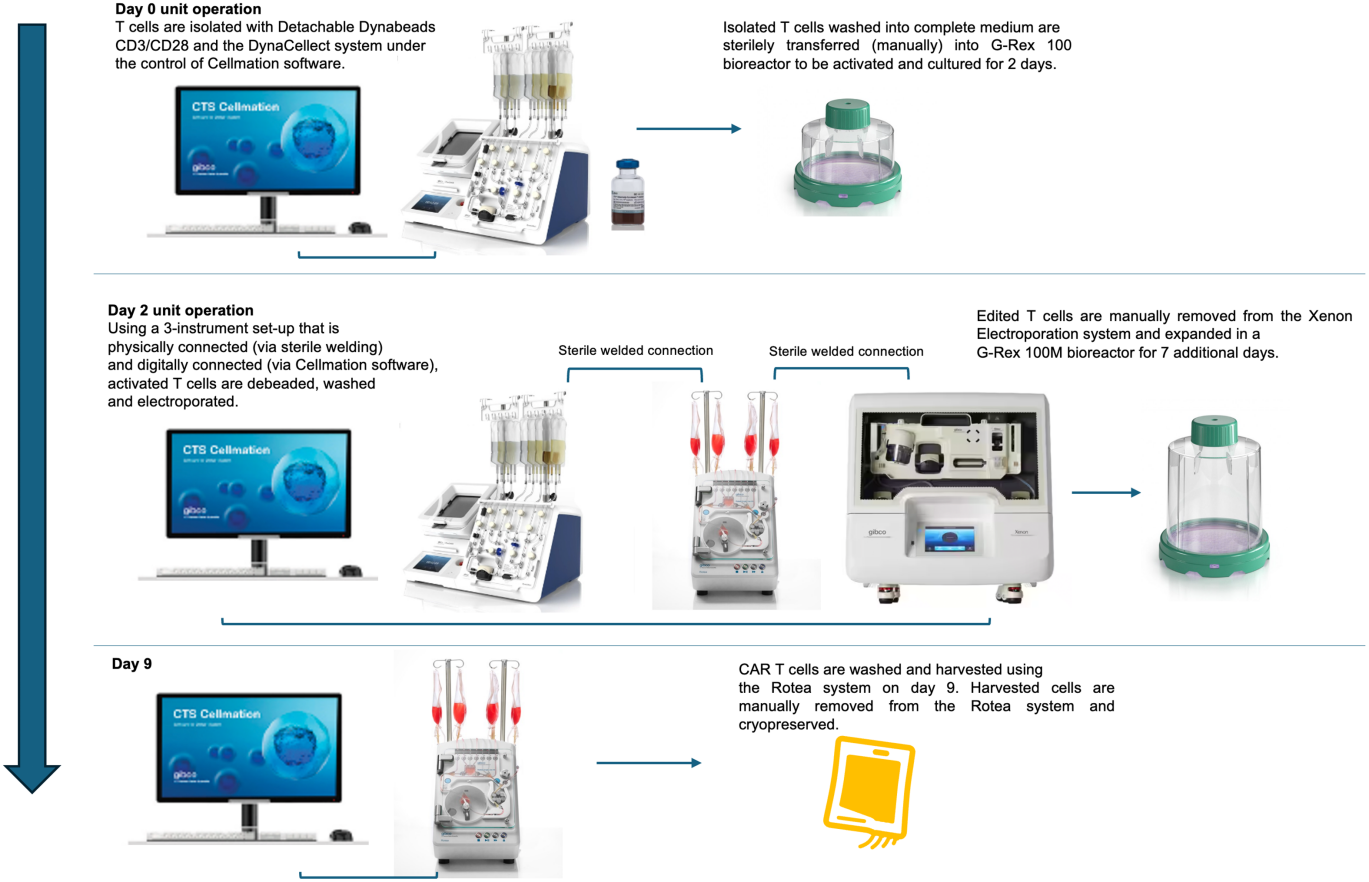
Overview of the semi‐automated CAR T‐cell manufacturing workflow using modular Thermo Fisher CTS instruments that can be connected both physically via sterile welding and digitally using the Gibco CTS Cellmation software.

CAR T‐cell therapy manufacturing is a multi‐day process with individual *unit operations* that span T‐cell isolation, T‐cell activation, gene modification, expansion and harvest (see Figure 1). For each unit operation, multiple modular Thermo Fisher Scientific Cell Therapy Systems (CTS™) instruments can be physically connected to execute the process in a flexible manner to accommodate a manufacturer's needs. Manual bioprocessing would require operators to finish one process on an instrument and then manually move the cells to the next instrument for processing. We can eliminate these manual steps by first physically integrating the instrument consumables for each step in the CAR T‐cell manufacturing process and then use automation software to control the overall process (Figure 1).

The CTS instruments are equipped with Open Platform Communications United Architecture (OPC‐UA), an industrial communication standard that allows an instrument to exchange data with other platforms or control systems.

Gibco™ CTS Cellmation™ software is an off‐the‐shelf automation solution that can help cell therapy manufacturers streamline their processes and address the challenges discussed. Through CTS Cellmation software, the instruments can be supervised, monitored and controlled automatically when instruments are configured to allow for remote operation via OPC‐UA.

By connecting multiple modular cell therapy instruments first physically, using welded polyvinyl chloride (PVC) tubing and then digitally, using CTS Cellmation software, manufacturers can create user‐specific batch recipes to control the flow of their manufacturing process. CTS Cellmation software was developed following Good Automated Manufacturing Process (GAMP) 5 methods to help ensure compatibility with Current Good Manufacturing Practice (CGMP)‐compliant processes.

Herein, we outline the method used to automate a cell therapy manufacturing workflow using CTS Cellmation software to control physically connected modular cell therapy instruments in the production of non‐viral CAR T cells.

## Results

The results demonstrate the successful generation of functional CAR T cells using a semi‐automated instrument workflow. The successful connection and control of the instruments using automation software present an exciting opportunity for process developers and manufacturers who want to reduce manual touchpoints in their cell therapy manufacturing process.

**Figure 2:**
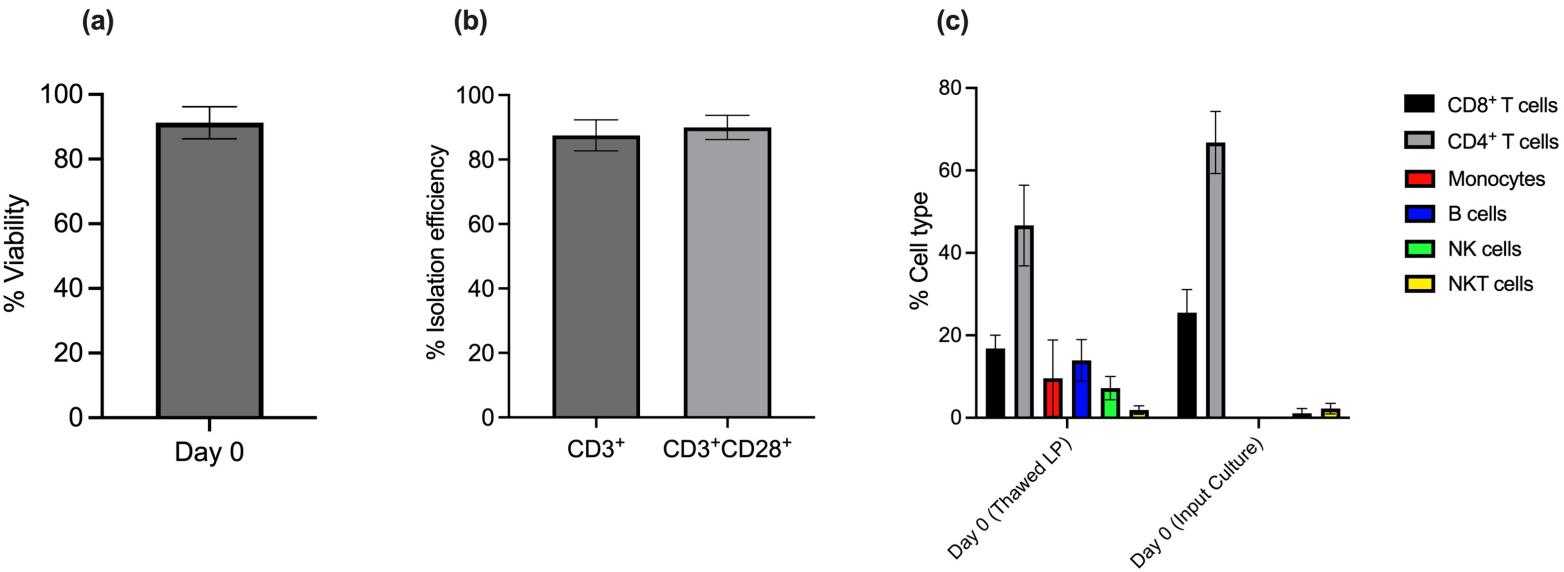
**(a)** Cell viability on the day of T‐cell isolation, day 0. **(b)** Isolation efficiency for CD3^+^ and CD28^+^ T cells on the day of T‐cell isolation. **(c)** Cell phenotypes on the day of T‐cell isolation. Data were generated from three different healthy donor leukopaks on three different dates.

The overall cell viability for each of the healthy donor cells used in these studies remained above 90% on the day of T‐cell isolation (day 0; Figure 2a). Furthermore, across the studies, isolation efficiency for both CD3^+^ and CD3^+^CD28^+^ T cells averaged above 90% (Figure 2b). The isolated fraction contained mainly CD4^+^ and CD8^+^ T cells on day 0. B cell and monocyte counts in the isolated fraction were negligible. The main contaminant cell type in the isolation fraction was NKT cells (CD3^+^CD56^+^) at a 2.27% mean percentage of the input culture (Figure 2c). Overall, these results demonstrate that the automated T‐cell isolation from thawed donor leukopaks (LP) was efficient.

**Figure 3:**
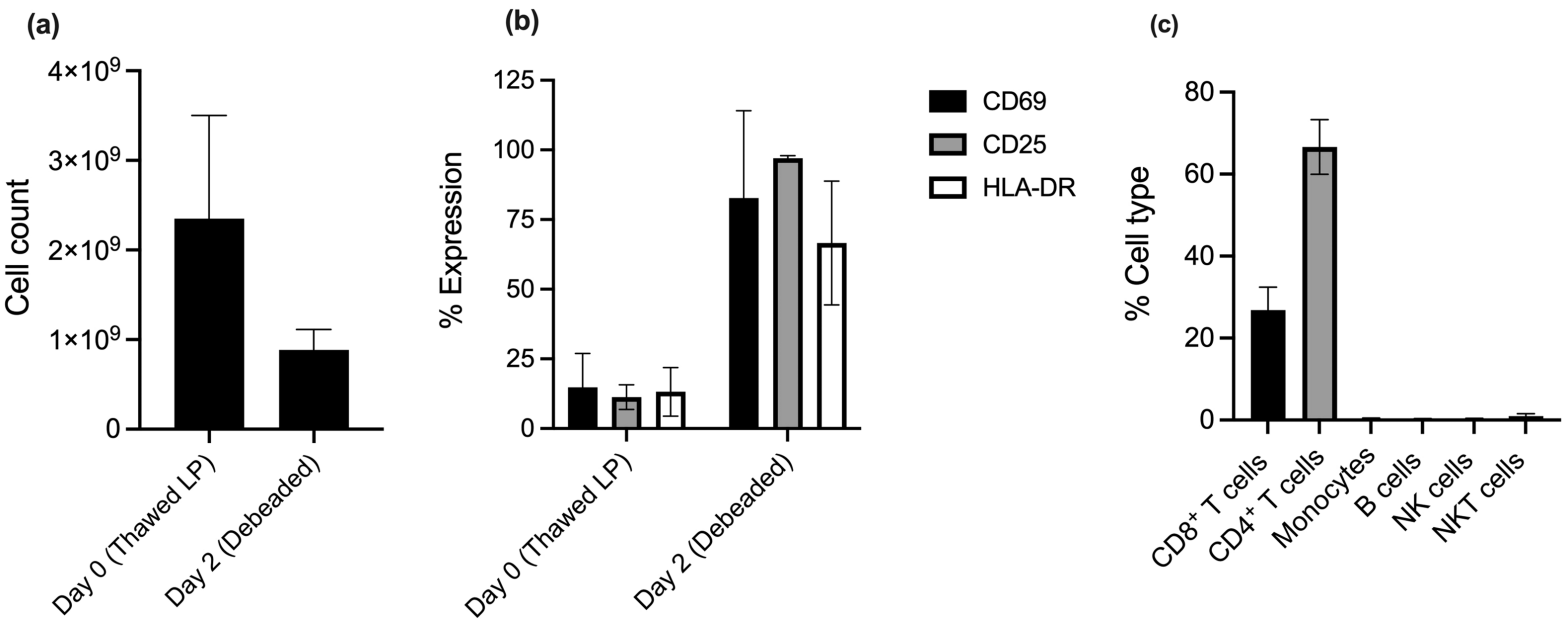
Parameters for starting leukopak material and on the day of bead removal were measured. **(a)** Total viable cell count. **(b)** T‐cell activation status and on both days. **(c)** Cell phenotype percentages present on day 2, post‐bead removal.

After 2 days of culture in a G‐Rex bioreactor, the Dynabeads CD3/CD28 beads were removed using the DynaCellect system. We recovered an average of 850 million viable cells on day 2 (Figure 3a). These cells were carried on for the next step of the manufacturing process. T‐cell activation markers CD69, CD25 and HLA‐DR were effectively induced by this timepoint (Figure 3b). The culture at this point maintained a higher proportion of CD4^+^ T cells (Figure 3c).

**Figure 4:**
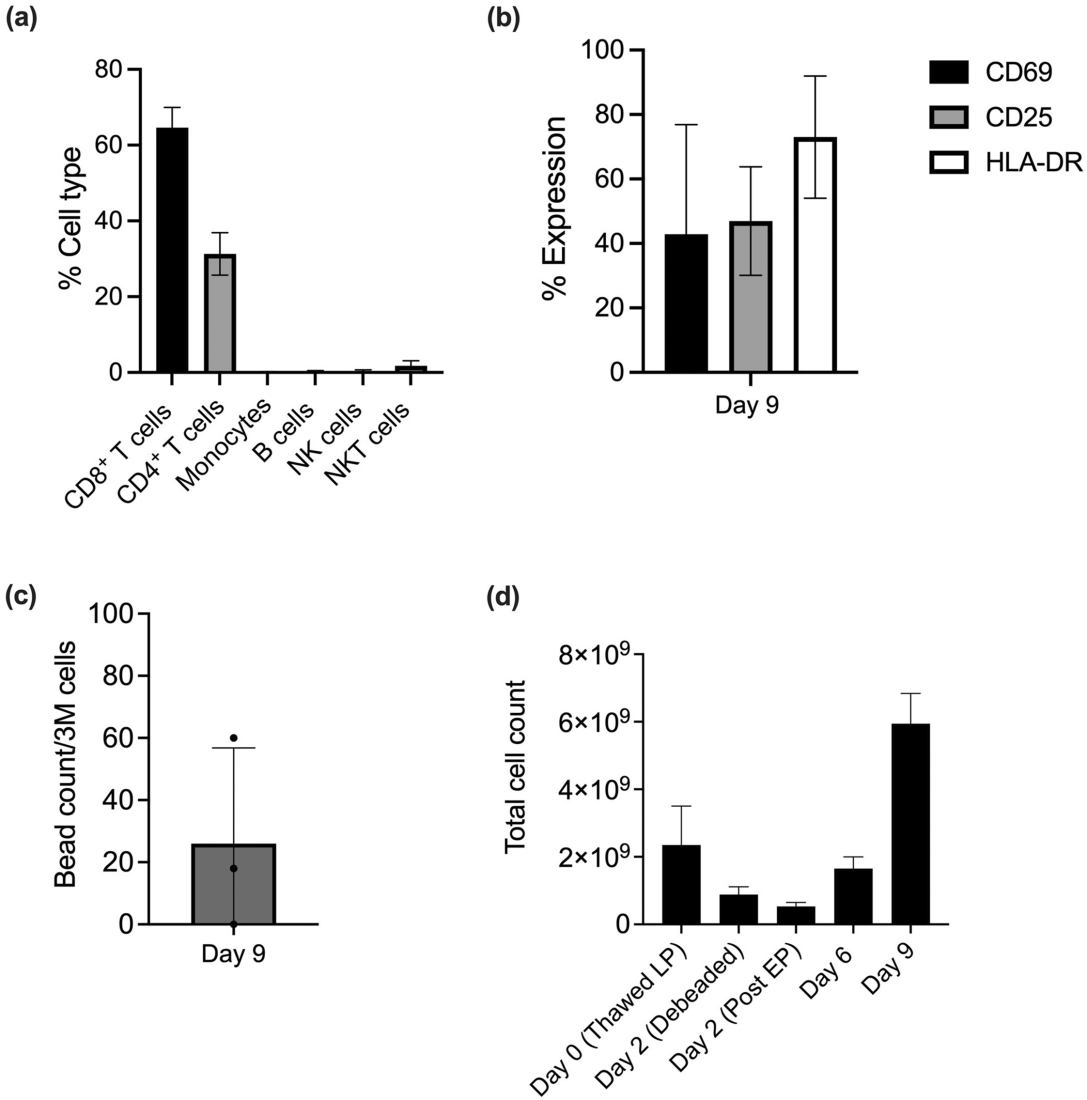
Parameters measured on day 9 (CAR T‐cell harvest day). **(a)** CAR T‐cell composition. **(b)** CAR T‐cell activation. **(c)** Residual magnetic bead counts. **(d)** Total cell count across donors from day 0 through day 9. EP, electroporation.

After electroporation and culture for seven additional days, however, the CAR T‐cell product had a significantly higher proportion of CD8^+^ T cells than of CD4^+^ T cells (Figure 4a). The CAR T cells were still activated at harvest (Figure 4b), and the number of residual Dynabeads was well below 100 per 3 million cells, which is compliant with US FDA CAR T‐cell manufacturing standards (Figure 4c). The average total viable cell count at each stage of the semi‐automated manufacturing process is shown in Figure 4d.

**Figure 5:**
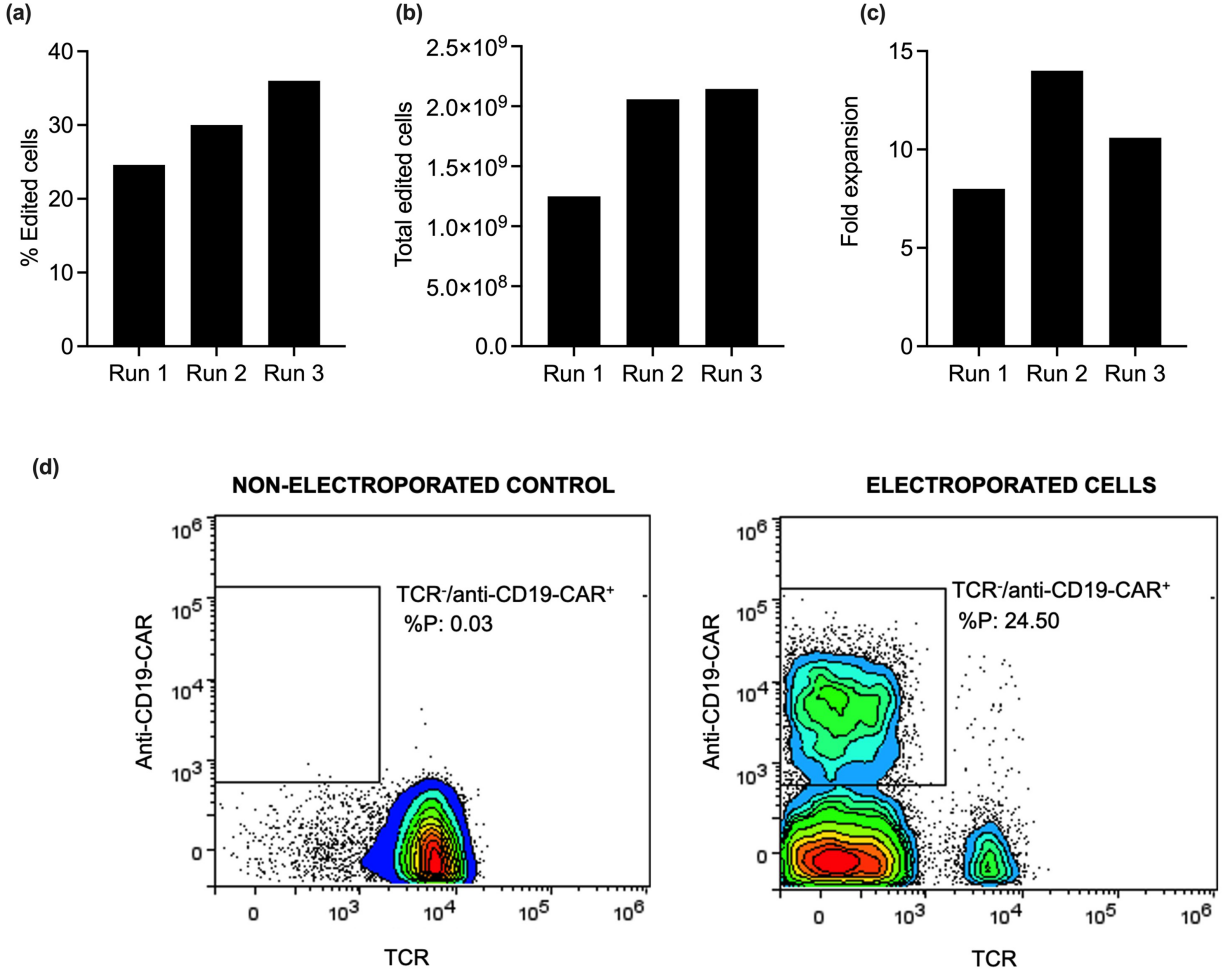
Flow cytometric analysis of the final CAR T‐cell product. **(a)** The percentage of edited T cells (compared to the overall viable cell count) for each donor/run. **(b)** Absolute numbers of CAR T cells at the end of each run. **(c)** CAR T‐cell fold expansion for each run. **(d)** Representative flow cytometry plots examining non‐electroporated/non‐edited T cells and electroporated/edited T cells.

When we assessed the number of fully edited (TCR^−^/anti‐CD19‐CAR^+^) T cells on the day of CAR T‐cell harvest, we found that the percentage of edited T cells ranged between 24% and 35% as shown in Figure 5a (each ‘Run’ represents CAR T‐cell manufacture from a distinct healthy donor). These percentages translated to an average of 1.6 billion CD19‐CAR^+^ T cells across donors (Figure 5b).

Fold expansion is measured by dividing the total number of viable cells at the end of expansion on day 9 by the total number of viable cells at day 2 post‐electroporation.

While we saw the cell numbers expand by 7‐fold, 14‐fold and 10‐fold, respectively (Figure 5c), across the three donors, these fold expansion numbers were lower than expected. Nonetheless, we achieved an adequate number of edited T cells that would be considered ‘clinically relevant’^3^ across the three studies.

Across the runs, we observed that our semi‐automated process that leverages electroporation and a CRISPR/Cas9 system allowed us to achieve greater than 90% TCR knockout. Of those cells, greater than 20% expressed the anti‐CD19 CAR gene (Figure 5d).

**Figure 6:**
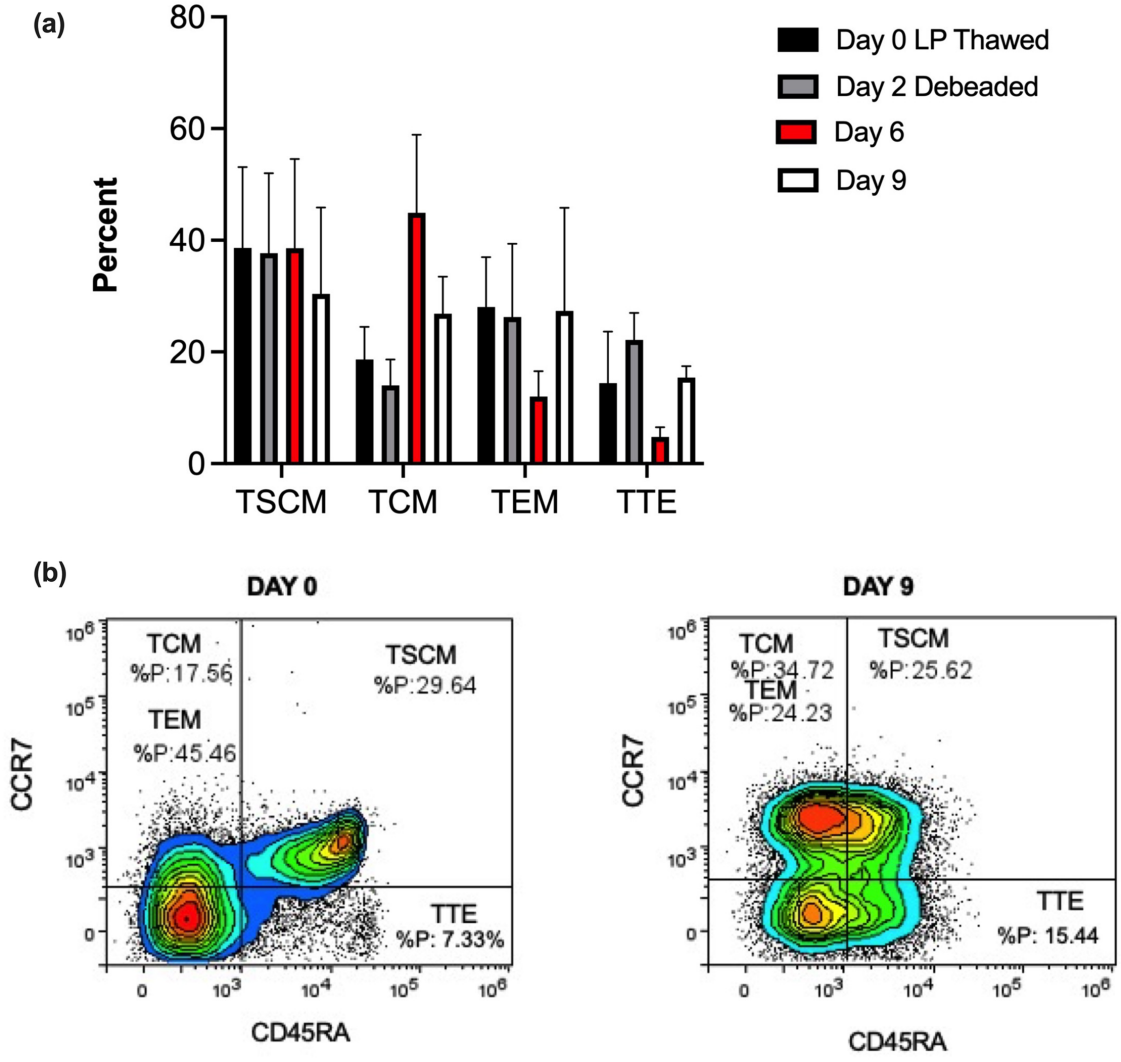
T‐cell memory phenotype through the CAR T‐cell manufacturing process. **(a)** The average percentage of the different memory cell phenotypes across the three donors. **(b)** A representative plot for one of the donors looking at the different memory cell phenotypes on day 0 following T‐cell isolation and on day 9 (the final CAR T‐cell product).

On average across donors, while we did not observe significant variations in the number of effector memory T cells (TEM), and terminal effector memory T cells (TTE), or stem cell‐like memory T cells (TSCM) from T‐cell isolation to CAR T‐cell harvest, we consistently observed an increase in the number of naïve central memory T cells (TCM) in the final CAR T‐cell product as shown in Figure 6a and b.

**Figure 7:**
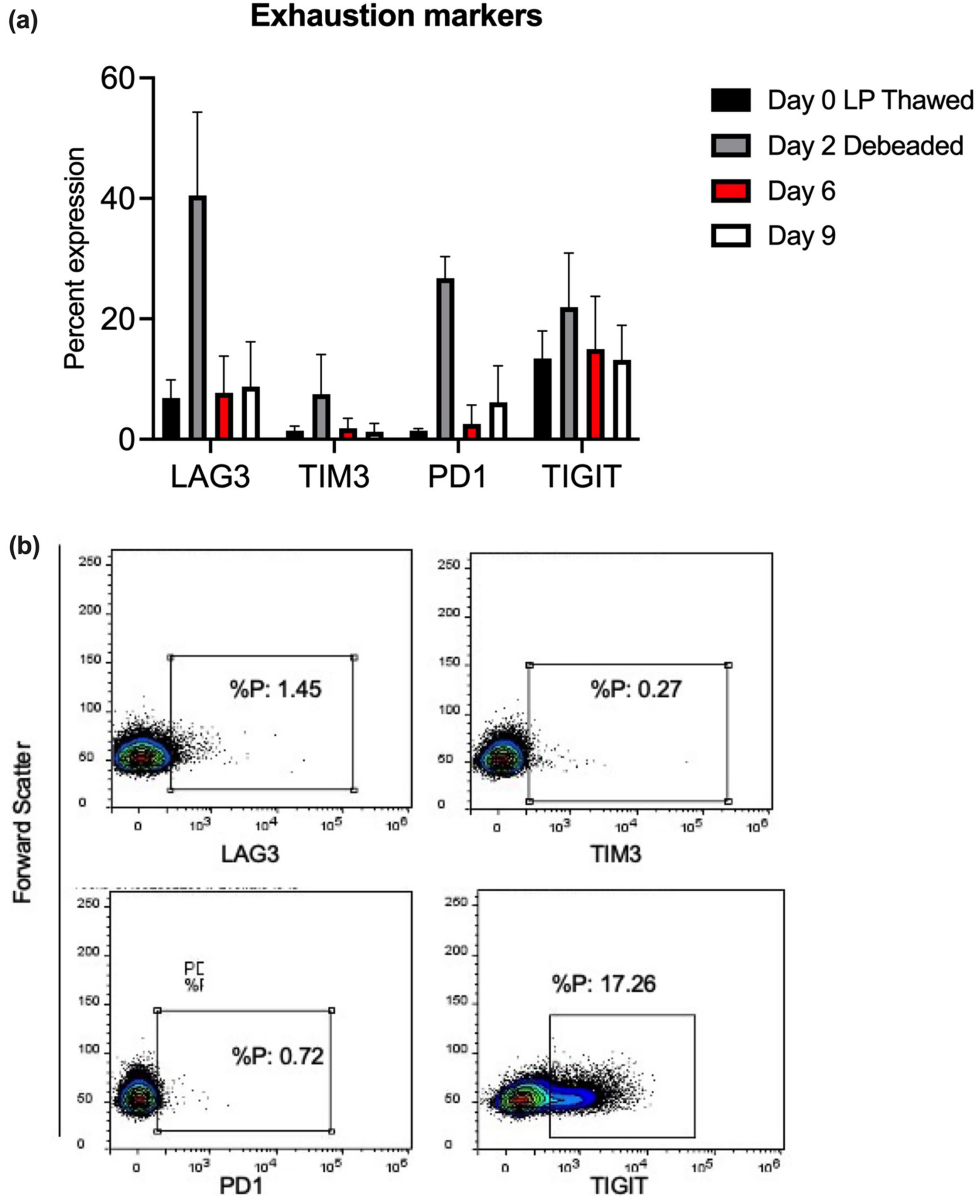
**(a)** The average expression of T‐cell exhaustion markers LAG3, TIM3, PD1 and TIGIT across the three final CAR T‐cell products from T‐cell isolation (day 0) through the day of CAR T‐cell harvest (day 9). **(b)** Representative flow cytometry plots for each T‐cell exhaustion marker in CAR T cells generated using the semi‐automated bioprocess on day 9.

The highest expression of exhaustion markers occurred on day 2 of the manufacturing process. This was likely because the cells were activated for 2 days prior to analysis. The expression of LAG3, TIM3 and PD1 was significantly lower at day 9 than at day 2. TIGIT expression levels remained the same throughout the manufacturing process (Figure 7).

**Figure 8:**
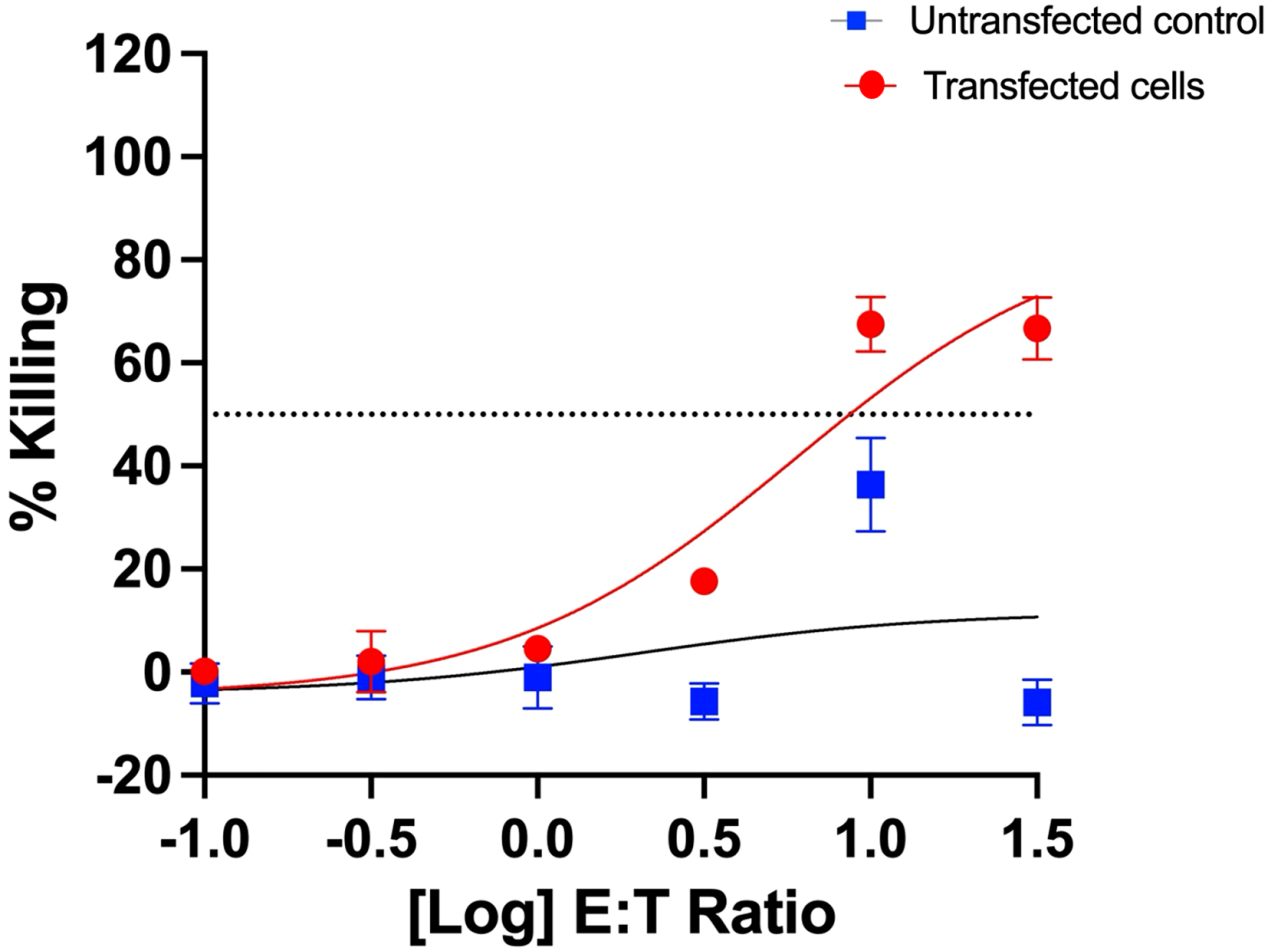
Cytotoxic activity of pre‐cryopreservation CAR T cells and untransfected control cells against Nalm6 cells was measured in triplicate for each donor. A representative cytotoxicity graph for one of the leukopak donors is shown here.

The median effective concentration (EC50) of CD19‐CAR^+^ T cells that was required to kill Nalm6 cell targets was significantly lower than the non‐electroporated controls as shown in Table 1. This observation is further demonstrated in Figure 8, implying that the CAR T cells produced using this end‐to‐end automated method are functional in recognising and killing target malignant cells.

**Table 1:** Cytotoxicity assay results

Run	CD19‐CAR kill assay (EC50 of E:T ratio)	No EP kill assay (EC50 of E:T ratio)
1	< 0.1	1.8
2	1.42	> 32
3	0.3	> 32

## Discussion

In this study, we sought to describe an end‐to‐end CAR T‐cell manufacturing method using Thermo Fisher's cell therapy manufacturing instruments under the control of an off‐the‐shelf automation software solution, CTS Cellmation. Overall, we observed that parameters like isolation efficiency, cell viability and T‐cell proportions over the manufacturing timeline were high and did not differ from previous observations that did not use the automation software (please refer to Supplementary figures 1–6).

The fact that the parameters measured throughout the semi‐automated process did not differ significantly from a manufacturing method that relied only on the modular instruments is important because drug developers and manufacturers who choose to adopt this process can rely on the fact that there will be no significant changes in their manufacturing process, or a loss in quality of CAR T cells while decreasing the variability and contamination risks that come with manual handling.

While this semi‐automated method involved some manual touchpoints, the automated day 2 process (for instance, from debeading through T‐cell editing) removed the following manual touchpoints that users would encounter if the instruments were not integrated using Cellmation:

1. Removing the debeaded T cells from the DynaCellect system
2. Remounting the debeaded T‐cell output onto the Rotea system for the pre‐electroporation wash
3. Removing the debeaded and washed T cells from the Rotea system
4. Setting up the cells on the Xenon system for electroporation

Each of these manual touchpoints presents an opportunity to introduce variability and contamination. Using the semi‐automated process, however, those manual touchpoints are eliminated. Additionally, because the instruments continuously share data with the Delta V network infrastructure that Cellmation is built on, using the semi‐automated process described in this paper helps to reduce the operator's time managing and monitoring individual instruments. Moreover, because the interactive Cellmation Software graphics are displayed on a single user interface for all instruments, real time information, operator messages, prompts, alarms and process critical parameters can all be accessed from one central location instead of multiple.

The DynaCellect, Rotea and Xenon systems can be used in research and development, process development and cell therapy manufacturing. However, for cell therapy manufacturing specifically, users of the instruments would need to purchase extra software to make these modular instruments 21 CFR part‐11 compliant. As part of this compliance, data and events that happen during the cell therapy manufacturing process need to be continually tracked and historised. This continual historisation and oversight prevents users from manipulating established variables as these actions will be captured in the historical record. Additionally, historisation allows for any errors encountered during the run to be traced back to a source point so that it can be rectified. So, apart from Cellmation linking the modular instruments digitally, it allows for data to be tracked and historised in a manner that is compliant with pharmaceutical regulations. In summary, while each of the instruments can be used as standalone instruments, using the Cellmation automation software can allow for seamless digital integration and oversight of the cell therapy manufacturing process.

For this study, we used a non‐viral method of modifying T cells that leveraged electroporation and the CRISPR–Cas9 system to deliver CD19‐CAR DNA to the TRAC locus.^4^ While the viral vector‐mediated method for gene delivery into T cells is a commonly used method in commercial cell therapy manufacturing, there are several limitations that are driving companies to switch to using non‐viral methods of gene delivery.

An important disadvantage of using virus in CAR T‐cell production is the increased risk of cancer development arising from insertional mutagenesis.^5^, ^6^ Additionally, virus‐derived DNA can impede CAR expression when the therapy is infused.^7^, ^8^ Moreover, virus manufacture comes at a high monetary cost.^9^ For these reasons, our lab has been focused on developing, optimising and using non‐viral electroporation tools in cell therapy process development alongside traditional viral methods.

The ability of CAR T cells to function optimally in a patient following infusion is related to their differentiation state. Naïve/central memory cells show the greatest anti‐cancer potency in previously published studies.^10^, ^11^ In this study, the proportion of TSCM remained unchanged throughout the manufacturing process, and we saw a consistent increase of TCM. Since TSCM is a source of developing TCM, this might suggest that CAR T cells produced using this automated process are highly potent against B‐cell malignancies.

While the fold expansion of T cells was lower than some of our previous experiments, we suspect a higher seeding density in the bioreactor on day 2 may have led to this. Nonetheless, a recent review found that across 74 studies, optimal clinical efficacy of CAR T cells was between 50 and 100 million cells.^3^ The number of CAR T cells at the end of all three experiments was significantly higher than this dose range and therefore could be relevant if this was manufactured for clinical use. Furthermore, these CAR T cells killed NALM6 cells effectively.

Harvested CAR T cells expressed low levels of exhaustion markers cells on day 9. Exhausted T cells often have an altered transcriptional profile that leads to a reduction in robust effector cell function.^10^ *In vivo*, T‐cell exhaustion is often related to an exposure to persistent antigen and/or inflammatory signals. In an *ex vivo* manufacturing process, T‐cell exhaustion could be triggered by operator handling and the components used in the isolation and expansion processes. The data presented herein suggest that automation may also help to keep exhaustion marker expression low due to reduced manual handling.

As more manufacturers begin to move to shorter cell therapy manufacturing workflows, it should be noted that, in another study,^12^ we demonstrated that the semi‐automated method using Cellmation software could be used to achieve a 24‐h lentiviral‐based manufacture of CAR T cells with low residual virus.

Further studies would be warranted to demonstrate full automation of cell therapy manufacturing using the modular instruments mentioned in this study. Additionally, the activity of CAR T cells produced using a semi‐ or fully automated process with these instruments in real‐world patients still needs to be explored. Nonetheless, the results herein show that we successfully generated CAR T cells using a semi‐automated instrument workflow. These findings could be beneficial to process development and manufacturing teams that require the flexibility of modular systems that can also be connected digitally during the cell therapy manufacturing process.

## Methods

### Experimental design

**Figure 9:**
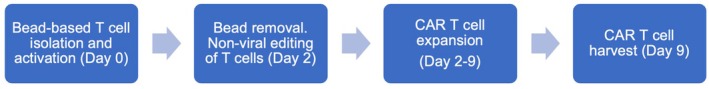
Flowchart showing the CAR T‐cell manufacturing process from day 0 (the day of T‐cell isolation and activation) to day 9 (the day of CAR T‐cell harvest).

Figure 9 provides an overview of the semi‐automated CAR T manufacturing method described herein.

The experiments described in this paper were performed on three different healthy donor blood samples on three different dates.

### Semi‐automated CAR T‐cell manufacturing workflow

#### Step 1: Setting up CTS Cellmation software for cell therapy instruments

All the experiments using these methods were performed in a Biosafety Level 2 laboratory.

CTS Cellmation Software (Cat. no. A59196, Thermo Fisher Scientific, Waltham, MA, USA) is powered by the Emerson DeltaV™ Distributed Control System (DCS). The CTS DynaCellect (Cat. no. A55867, Thermo Fisher Scientific), Rotea (Cat. no. A44769, Thermo Fisher Scientific) and Xenon (Cat. no. A50301, Thermo Fisher Scientific) were controlled using DeltaV software batch recipes.

For our experiments, communication between the DynaCellect, Rotea and Xenon systems was established via a Cat 5e/Cat 6 cable that was used to connect the instruments to the DeltaV system hardware.

**Figure 10:**
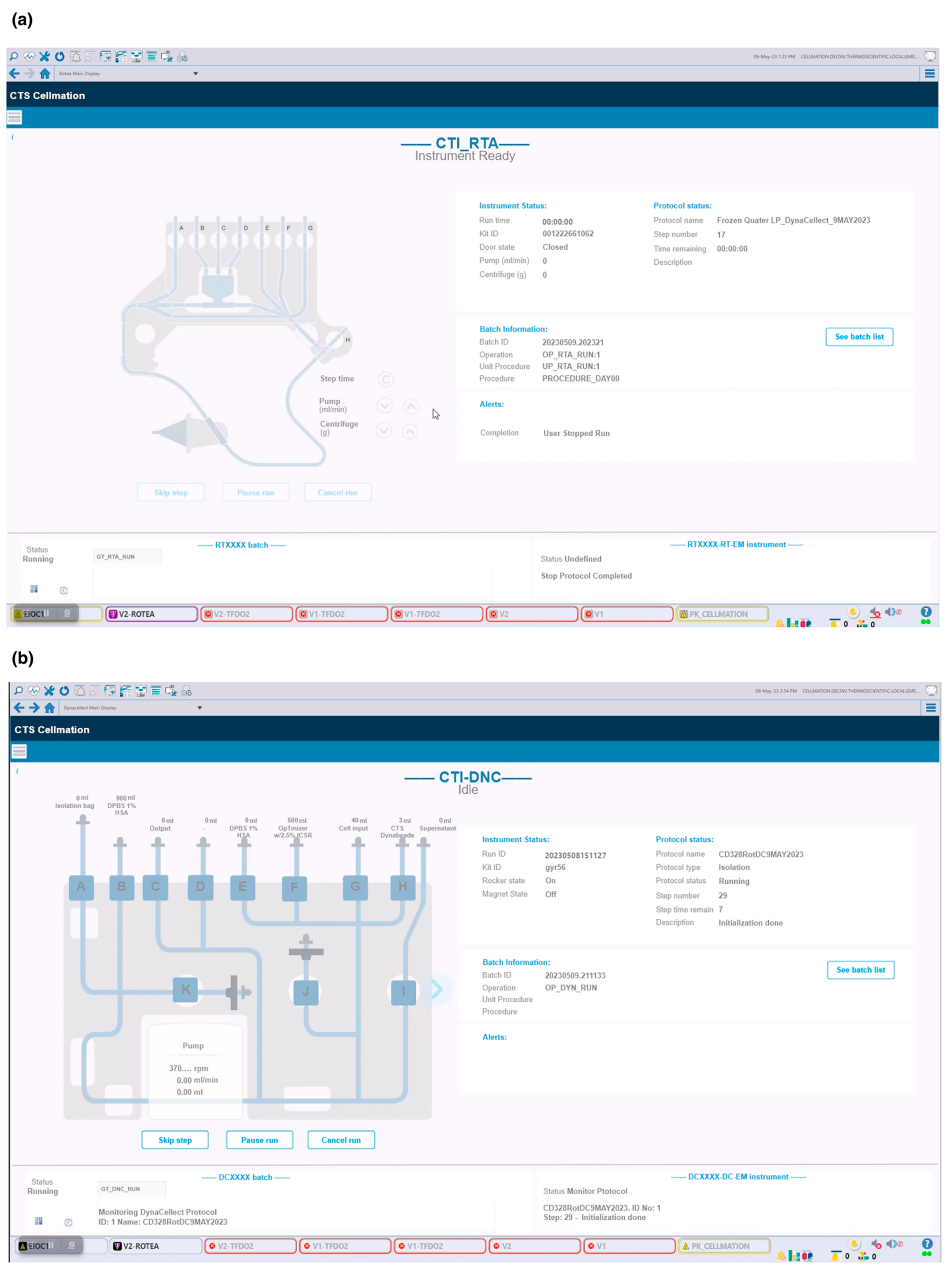
CTS Cellmation graphical user interface for the **(a)** CTS Rotea and **(b)** CTS DynaCellect systems.

Signals from the cell therapy instruments were captured in the equipment control modules in the Cellmation software. The parameters assessed in each of these modules were visualised on a graphical user interface to show the progress of the protocols as shown in the screenshot examples in Figure 10.

The equipment modules performed supervisory control of the cell therapy instruments primarily by initiating protocols and by controlling the steps in a protocol to completion.

#### Step 2: T‐cell isolation (day 0 unit operation)

A quarter leukopak (approximately, 65 mL) was thawed using the ThawSTAR CB Automated Thawing System (Cat. No. AST‐90000, BioLife Solutions Inc., Bothell, WA, USA). Once the bag was thawed, 1‐mL samples for each experiment were collected into clean microcentrifuge tubes for immunophenotyping analysis.

T cells were isolated from thawed donor leukopaks using the Gibco CTS Detachable Dynabeads™ CD3/CD28 (Cat. No. A56996, Thermo Fisher Scientific), the CTS DynaCellect Cell Isolation Kit (Cat. No. A52300, Thermo Fisher Scientific) and the CTS DynaCellect Magnetic System under the control of the CTS Cellmation module designed for the DynaCellect system.

The bag setup for T‐cell isolation on day 0 using the DynaCellect System is shown in Table 2.

**Table 2:** T‐cell isolation on day 0 using the DynaCellect system

Port	Bag/Content
A	DynaCellect isolation bag
B	DPBS with 1% human serum albumin HSA for B‐E wash loop (900 mL)
C	Output bag (1000 mL)
D	Not used/no bag attached
E	DPBS with 1% HSA for B‐E wash loop (same bag as on B)
F	CTS OpTmizer Pro for export into output bag (500 mL)
G	Thawed leukopak cells (cell input bag)
H	CTS Dynabeads CD3/CD28 beads in conical bag
I	Bag for supernatant/waste fraction (1000 mL)

The output T cells were isolated directly into CTS OpTmizer™ Pro (Cat. No. A4966101, Thermo Fisher Scientific) supplemented with ICSR (Immune Cell Serum Replacement) (Cat. No. A2596102, Thermo Fisher Scientific) CTS GlutaMAX‐I (Cat. No. A1286001, Thermo Fisher Scientific), L‐Glutamine (Cat. No. 25030081, Thermo Fisher Scientific) and PeproGMP human recombinant IL2 (Cat. No. GMP200‐02, Thermo Fisher Scientific). After isolation, T cells were transferred into and cultured in a G‐Rex 100 bioreactor vessel (Cat. No. 80500, Wilson Wolf, Saint Paul, MN, USA) for 2 days in the Thermo Scientific HeraCell Vios CR 250i CO_2_ Incubator (CTS series) at 37°C and 5% CO_2_. At each stage of the cell therapy manufacturing process, cell samples were collected for analysis.

Isolation efficiency for the T‐cell isolation process was calculated using the following formula:

$$\text{Isolation efficiency}=\left(1-\frac{\text{Supernatant}\;T\;\text{cells}}{\text{Input}\;T\;\text{cells}}\right)\;x\;100.$$

#### Step 3: Bead removal and non‐viral gene editing (day 2 unit operation)

The consumables for the DynaCellect, Rotea and Xenon systems were connected to each other physically via welded PVC tubing.

After the single‐use consumables for the CTS DynaCellect system, the CTS DynaCellect Isolation kit, were unboxed, the following bags were prepared and welded onto the CTS DynaCellect Bead Removal Kit (see Table 3 for port setup).

**Table 3:** Day 2 setup for the bead removal using the DynaCellect System

Ports	Bag/Content
A	DynaCellect isolation bag
B	CTS DPBS with 1% HSA (B‐E loop) (400 mL)
C	Empty output bag (1 L) (welded to Rotea system)
D	Not used/no bag attached
E	CTS DPBS with 1% HSA (B‐E loop) (400 mL)
F	CTS Detachable Dynabeads release buffer (125 mL)
G	Input cells in bag (500 mL)
H	Not used/no bag attached
I	Not used/no bag attached

*The wash buffer bag* was a sterile 600‐mL blood bag that was spiked to enable filling via Luer‐lock. It was filled using a 60‐mL Luer‐lock syringe (Cat. No. 14955455, Thermo Fisher Scientific) with 400 mL of DPBS (Cat. No. A1285602, Thermo Fisher Scientific) supplemented with 1% Human Serum Albumin (Cat. No. ALB064302, Octapharma USA, Paramus, NJ, USA).

*The release buffer bag* was a sterile 300‐mL bag filled with 125 mL of Detachable Dynabeads Release Buffer (Cat. No. A5588303, Thermo Fisher Scientific). *The input bag* was a 1‐L bag containing the activated, cultured T cells. Instead of an empty *output bag* that is normally mounted on the DynaCellect instrument, the ‘output bag’ for this process was mounted on the Rotea system (‘input bag’ for the subsequent Rotea wash process). This bag was sterilely welded using PVC tubing to the C port on the DynaCellect Isolation Kit.

Next, the CTS Rotea Single‐Use Kit (Cat. No. A49313, Thermo Fisher Scientific) was unboxed. The following bags were prepared to be welded onto the kit using the port setup displayed in Table 4.

**Table 4:** Day 2 setup for the Rotea Single‐Use kit

Ports	Bag/Content
A	1000‐mL bag for waste
B	300‐mL bag for 200 mL DPBS/1% HSA
C	Gibco CTS Xenon genome editing buffer (bag)
D	Cell input loop with port G (welded to C line on the DynaCellect instrument)
E	2‐mL payload in 10‐mL sampling pouch
F	Empty
G	Cell input loop with port D (welded to C line on the DynaCellect instrument)
H	Direct weld PVC line to Xenon

As noted above, instead of a 1‐L output bag that is typically mounted on the DynaCellect instrument, the output bag for this process was mounted on the Rotea system.

The *wash buffer bag* was a 300‐mL bag containing 200 mL of buffer (DPBS with 1% HSA).

Next, an *empty 1‐L waste bag* for collecting waste from the bead removal and washing processes was prepared. The CTS Xenon *Genome Editing Buffer* (Cat. No. A4998002, Thermo Fisher Scientific) comes in a 100‐mL bag. It requires a PVC‐Cflex adapter to be welded to Rotea Single‐Use Kit (Cat. No. CT‐014‐CVT, Charter Medical LLC, Winston‐Salem, NC, USA).

The required *payload* as shown in Table 3 was prepared; 2 mL of the payload was added to the sampling pouch with an added spike‐male adapter. The purpose here was to add a section of PVC tubing to sterile weld to the Rotea Kit on port E.

Using PVC tubing, the H port of the Rotea Single‐Use kit was welded to the tubing attached to the CTS Xenon Multishot Electroporation Cartridge (Cat. No. A50306, Thermo Fisher Scientific).

Once all the connections were made, each instrument consumable was mounted onto the respective instrument.

Once the debeaded cells underwent washing on the Rotea System, the washed T cells, the payload and Genome Editing Buffer were transferred to the CTS Xenon Multishot Electroporation Cartridge.

The physically connected instruments were used to perform the combined bead removal‐wash‐electroporation module under the control of CTS Cellmation software (Figure 1).

For the experiments in this paper, anti‐CD19 CAR was transfected into T cells using the CTS Xenon Electroporation System (electroporation parameters are shown in Table 5) on day 2 following bead removal and cell washing.

**Table 5:** Electroporation conditions on the CTS Xenon system

Voltage	Pulse width	Number of pulses	Pulse interval
2300 V	3 ms	4	500 ms

The components of the payload delivered to the activated T cells included CTS TrueCut Cas9 Protein (Cat. No. A45220, Thermo Fisher Scientific), Invitrogen TrueGuide Synthetic guide RNA (Custom, Thermo Fisher Scientific) and double‐stranded anti‐CD19 CAR DNA (Custom, LineaRx, Stonybrook, NY, USA) (as shown in Table 6).

**Table 6:** Payload component concentrations per millimetre

Reagent	Amount per mL (50 × 10^6^ cells)	Stock concentration	Final concentration
CAS9 HiFi	12 μL	10 mg mL^−1^	120 ng mL^−1^
gRNA (TRAC)	10 μL	320 mM	3.2 mM
dsDNA (CD19 CAR)	80 μL	2 mg mL^−1^	160 μg mL^−1^

Post‐electroporation, the edited T cells were cultured and expanded in CTS OpTmizer Pro SFM supplemented with ICSR, CTS GlutaMAX‐I and L‐Glutamine in a G‐Rex‐100 M closed system bioreactor for 7 days at 37°C. The culture was fed with fresh complete media every 2–3 days.

#### Step 4: CAR T‐cell wash and harvest (day 9 unit operation)

On day 9, CAR T cells were washed and harvested using the Rotea system (see Table 7 for the high‐speed wash and harvest setup).

**Table 7:** Wash and harvest on the Rotea system

Ports	
A	1000‐mL bag for waste
B	200‐mL wash buffer (DPBS/2% HSA)
C	Empty
D	CAR T‐cell input loop with G
E	Empty
F	Empty
G	CAR T‐cell input loop with D
H	CryoStore bag/output with equal intended volume of CS10

The G‐Rex bioreactor containing the CAR T cells was removed from the incubator and into a biosafety cabinet. A serological pipette was used to resuspend the CAR T‐cell culture. A sterile 60‐mL syringe was then used to collect the CAR T cells from the G‐Rex bioreactor. This was then used to fill the 1000‐mL transfer bag via a female luer port.

The 1000‐mL bag containing the CAR T cells (input), an empty 1000‐mL transfer bag (for waste), a bag containing 200 mL wash buffer (DPBS/2% HSA) and a CryoStore bag (Cat. No. CS250N, Origen Biomedical, Austin, Texas) with an equal volume of CS10 Freeze Medium (Cat. No. 210102, BioLife Solutions Inc, Bothell, WA, USA) of the intended output volume were welded to the CTS Rotea Single‐Use Kit.

Once the CAR T cells were washed on the Rotea system, they were collected into the 250‐mL CryoStore bag. Using a sterile port, 50 mL of CS10 Freeze Medium was added to the harvested CAR T cells to make a final density of about 10–20 million cells mL^−1^. This bag was transferred to the CTS CryoMed Controlled Rate freezer (Cat No. TSCM17PA, Thermo Fisher Scientific). Once the storage temperature reached −80°C, the CryStore bag was transferred into liquid nitrogen for long‐term storage.

The results are representative of three independent experiments with material from three different healthy donor leukopaks.

### Cell analysis

#### Flow cytometry

Cell samples were collected and centrifuged at 400× *g* for 5 min at room temperature to remove the cell culture or wash buffer medium. The supernatant fraction was removed, and the sample was washed with 1 mL eBioscience™ flow cytometry staining buffer (Cat. No. 00‐422‐26, Thermo Fisher Scientific).

The samples were centrifuged again at 400× *g* for 5 min at room temperature followed by removal of the supernatant fraction. The cell sample was then resuspended in a 20% solution of eBioscience Anti‐Human Fc Receptor Binding Inhibitor Polyclonal Antibody, (Fc block) (Cat. No. 14‐9161‐73, Thermo Fisher Scientific) diluted in flow cytometry staining buffer. Cell samples were plated into 96‐well round bottom plates; ~0.3 × 10^6^ cells were plated per well in a total volume of 100 μL. Each sample was divided into three groups: stained, unstained plus viability indicator dye and unstained. Incubation in the 20% Fc block solution spanned 15 min at 4°C in the dark. After this, the stained groups were incubated in freshly made antibody cocktails for 15 min at 4°C in the dark.

Then, the cells were centrifuged again at 400× *g* for 5 min at room temperature. The supernatant fraction was removed, and the samples were washed with 200 μL flow cytometry staining buffer. This was followed by centrifugation at 400× *g* for 5 min at room temperature. The supernatant fraction was removed, and the stained and unstained plus viability indictor dye groups were resuspended in 200 μL of a 1:500 dilution of the viability dye. Fixable dyes were incubated for 30 min at room temperature protected from light. The unstained control groups were resuspended in 200 μL of FSS. This was followed by acquisition of the samples on the Attune CytPix flow cytometer (Cat. No. A48664, Thermo Fisher Scientific) with CytKick™ MAX autosampler (Cat. No. A38976, Thermo Fisher Scientific).

Data were acquired with Attune Cytometric Software v6.0.0 and analysed using FlowLogic 8.6 (Inivai Technologies, Victoria, Australia). Results were visualised using GraphPad Prism v9.3.1 (Dotmatics, Boston, MA, USA).

Flow cytometry antibody panels used are outlined in Tables 8, 9, 10, 11. The gating strategies for all flow cytometry experiments are shown in Supplementary table 1.

**Table 8:** Flow cytometry antibody panel for general phenotyping analysis

Cell marker	Product number
CD3	Invitrogen (47‐0036‐42)
CD4	Invitrogen (67‐0049‐42)
CD8	Invitrogen (56‐0087‐42)
CD14	Invitrogen (25‐0149‐42)
CD45	Invitrogen (63‐9459‐42)
CD56	Invitrogen (12‐0567‐42)
CD19	Invitrogen (46‐0198‐42)
CD28	Invitrogen (17‐0289‐42)
CD2	Invitrogen (78‐0029‐42)
SYTOX Blue dead cell stain	Invitrogen (S34857)

**Table 9:** Flow cytometry antibody panel for analysing T‐cell activation status

Cell marker	Product number
CD3	Invitrogen (47‐0036‐42)
CD4	Invitrogen (67‐0049‐42)
CD8	Invitrogen (56‐0087‐42)
CD25	Invitrogen (61‐0257‐42)
CD69	Invitrogen (46‐0699‐42)
HLA‐DR	Invitrogen (63‐9956‐42)
CD2	Invitrogen (78‐0029‐42)
SYTOX Blue dead cell stain	Invitrogen (S34857)

**Table 10:** Flow cytometry antibody panel for analysing T‐cell memory phenotypes

Cell marker	Product number
CD45RA	Invitrogen (11‐9979‐42)
CD62L	Invitrogen (17‐0629‐42)
CD8	Invitrogen (A15448)
TCR α/β	Invitrogen (48‐9986‐42)
CCR7 (CD197)	Invitrogen (61‐1979‐42)
CD4	Invitrogen (25‐0049‐42)
V5‐Tag	Invitrogen (12‐6796‐42)
LIVE/DEAD fixable aqua dead cell stain kit	Invitrogen (L34966)

**Table 11:** Flow cytometry antibody panel for analysing T‐cell exhaustion markers

Cell marker	Product number
CD223 (LAG‐3)	Invitrogen (56‐2239‐42)
CD279 (PD‐1)	Invitrogen (64‐9969‐42)
CD8	Invitrogen (A15448)
CD4	Invitrogen (25‐0049‐42)
LIVE/DEAD fixable aqua dead cell stain kit	Invitrogen (L34966)

#### Residual bead quantification

9 × 10^6^ CAR T cells were collected for residual bead quantification, and 3 × 10^6^ cells were used for each of three technical replicates. CAR T‐cell samples were centrifuged at 21 000× *g* for 5 min at room temperature using a table‐top centrifuge. Centrifuged samples were placed on the DynaMag™‐2 (Cat. No. 12321D, Thermo Fisher Scientific) for 1 min. The supernatant was removed without disturbing the Detachable Dynabeads CD3/CD28 beads or cell pellet. The microcentrifuge tube was removed, and the cell‐bead pellet was resuspended in 1 mL of 1× RIPA buffer (Cat. No. XG348655, Thermo Fisher Scientific) and then incubated in 10 μL DNase 1 (Cat. No. 18047‐019, Thermo Fisher Scientific) for 20 min at 37°C. Fifty microlitres of Proteinase K (Cat. No. 25530049, Thermo Fisher Scientific) was added after this and incubated for an additional 20 min at 65°C. The samples were then centrifuged at 21 000× *g* for 5 min at room temperature. The samples were placed on the DynaMag‐2 for 1 min, and then, the supernatant was removed. The samples were vortexed for 5 s and then pulse centrifuged using a table‐top centrifuge to collect all the liquid to the bottom of the tube. The volume of remaining suspension was brought up to 12 μL with 1× RIPA buffer. The sample was pulse centrifuged again, and then, 12 μL of sample was loaded into Kova™ Glasstic slide chambers (Cat. No. 87146, Kova Plastics, Garden Grove, CA, USA). The Invitrogen EVOS™ XL Core bright‐field microscope (Cat. No. AMEX1200, Thermo Fisher Scientific) was used to count the beads in each chamber. The formula below was used to calculate the amount residual beads per 3 × 10^6^ T cells post‐debeading.

$$\begin{array}{l} \text{Number of Dynabeads}\;\mathrm{per}\;3\;x\;10^{6}\;\text{cells}=\\ \;\begin{array}{l} \frac{\text{Total Dynabeads counted in}\;x\;\text{grids}}{\text{Number of grids counted}}*\;90*12\;\mathrm{\mu L}\;\text{of sample loaded} \end{array} \end{array}$$

Data were analysed and visualised using GraphPad Prism v9.3.1 (350).

#### Cytotoxicity assay

The cytotoxicity assay was performed using the Promega One‐Glo™ Luciferase Assay System (Cat. No. E6120, Promega Corporation, Madison, WI, USA).

Following culture, NALM6 cells, unmodified control cells and CAR T cells were centrifuged at 400× *g* for 5 min. Spent media was discarded.

The 2 × 10^6^ NALM6 cells were resuspended in 10 mL of warmed RPMI 1640 medium (Cat. No. 11835030, Thermo Fisher Scientific), while 1 × 10^6^ of the control cells and CAR T cells were each resuspended in 1 mL of warmed RPMI.

Using a multichannel pipettor, 100 μL of warmed RPMI medium was added to each row of two round‐bottomed 96‐well plates except for row B. Using a p200 pipettor, 100 μL of resuspended control cells and CAR T cells was added to the wells in row A in triplicate (in the appropriate plate); 200 μL of either the control cells or CAR T cells was added to row B. A multichannel pipettor was used to collect 100 μL of cells from row B and used to create a serial dilution from rows C through G. The remaining 100 μL from the G row was discarded. The number of effector cells used was calculated using the percentage of CAR‐expressing T cells in culture.

Next, 100 μL of NALM6 cells was added to each of the wells except row A.

The unmodified control cells and CAR T cells were incubated with the NALM6 cells overnight in the 96‐well plates at 37°C.

Following incubation, the cells in the 96‐well plates were resuspended by pipetting up and down three to four times. One hundred millilitres of resuspended cells was collected from each well into the wells of new white flat‐bottomed 96‐well plates. The ONE‐Glo Luciferase Assay reagent was prepared according to manufacturer instructions. One hundred microlitres of the reagent was added to each well in the flat‐bottomed 96‐well plates. These plates were covered and incubated at room temperature for 15 min.

The cytotoxicity assay was performed in triplicate for each of the three final CAR T‐cell products generated from the three different donors.

A SpectraMax™ plate reader (Molecular Devices, San Jose, CA, USA) was used to measure luminescence.

## Author contributions

**Jason Isaacson:** Conceptualization; data curation; formal analysis; investigation; methodology; project administration. **Prajakta Bhanap:** Investigation; software. **Nicholas Putnam:** Data curation; investigation; methodology. **Jasmine Padilla:** Formal analysis; investigation. **Nujhat Fatima:** Investigation. **Max Dotson:** Investigation. **Danny Hayoun:** Investigation. **Moloud Ahmadi:** Data curation; resources. **Gertrude Nonterah:** Writing – original draft; writing – review and editing. **Yongchang Ji:** Conceptualization; project administration; supervision.

## Conflict of interest

The authors declare no conflict of interest.

## Supporting information


**Supplementary figures** **1–6**

**Supplementary table** **1**

## Data Availability

The data that support the findings of this study are available from the corresponding author upon reasonable request.

## References

[1] Sharma P , Kasamon YL , Lin X , Xu Z , Theoret MR , Purohit‐Sheth T . FDA approval summary: Axicabtagene Ciloleucel for second‐line treatment of large B‐cell lymphoma. Clin Cancer Res 2023; 29: 4331–4337. 37405396 10.1158/1078-0432.CCR-23-0568 PMC10767767

[2] Neelapu SS , Dickinson M , Munoz J *et al*. Axicabtagene ciloleucel as first‐line therapy in high‐risk large B‐cell lymphoma: the phase 2 ZUMA‐12 trial. Nat Med 2022; 28: 735–742. 35314842 10.1038/s41591-022-01731-4 PMC9018426

[3] Rotte A , Frigault MJ , Ansari A , Gliner B , Heery C , Shah B . Dose‐response correlation for CAR‐T cells: a systematic review of clinical studies. J Immunother Cancer 2022; 10: e005678. 36549782 10.1136/jitc-2022-005678 PMC9791395

[4] Eyquem J , Mansilla‐Soto J , Giavridis T *et al*. Targeting a CAR to the *TRAC* locus with CRISPR/Cas9 enhances tumour rejection. Nature 2017; 543: 113–117. 28225754 10.1038/nature21405 PMC5558614

[5] Michieletto D , Lusic M , Marenduzzo D , Orlandini E . Physical principles of retroviral integration in the human genome. Nat Commun 2019; 10: 575. 30718508 10.1038/s41467-019-08333-8 PMC6362086

[6] Russo‐Carbolante EM , Picanço‐Castro V , Alves DC *et al*. Integration pattern of HIV‐1 based lentiviral vector carrying recombinant coagulation factor VIII in Sk‐hep and 293T cells. Biotechnol Lett 2011; 33: 23–31. 20812025 10.1007/s10529-010-0387-5

[7] Atianand MK , Fitzgerald KA . Molecular basis of DNA recognition in the immune system. J Immunol 2013; 190: 1911–1918. 23417527 10.4049/jimmunol.1203162 PMC3660856

[8] Tao J , Zhou X , Jiang Z . cGAS‐cGAMP‐STING: the three musketeers of cytosolic DNA sensing and signaling. IUBMB Life 2016; 68: 858–870. 27706894 10.1002/iub.1566

[9] Gándara C , Affleck V , Stoll EA . Manufacture of third‐generation lentivirus for preclinical use, with process development considerations for translation to good manufacturing practice. Hum Gene Ther Methods 2018; 29: 1–15. 29212357 10.1089/hgtb.2017.098 PMC5806069

[10] Ghassemi S , Durgin JS , Nunez‐Cruz S *et al*. Rapid manufacturing of non‐activated potent CAR T cells. Nat Biomed Eng 2022; 6: 118–128. 35190680 10.1038/s41551-021-00842-6 PMC8860360

[11] Wherry EJ , Kurachi M . Molecular and cellular insights into T cell exhaustion. Nat Rev Immunol 2015; 15: 486–499. 26205583 10.1038/nri3862 PMC4889009

[12] Ahmadi M , Putnam N , Dotson M *et al*. Accelerating CAR T cell manufacturing with an automated next‐day process. Curr Res Transl Med 2024; 73: 103489. 39705851 10.1016/j.retram.2024.103489

