# Supplementary material for: Enhancing CAR T‐cell therapy manufacturing efficiency through semi‐automated bioprocessing
Source: Clin Transl Immunology. 2025 Jun 2;14(6):e70025. doi: 10.1002/cti2.70025 (PMC12129705; doi:10.1002/cti2.70025)
Supplement: Supplementary file 1 — Supplementary figures 1–6 [file CTI2-14-e70025-s001.docx]

**Supplementary figures**

**Supplementary figure 1:** Initial leukopak (LP) and isolated fraction (day 2) cell compositions performed on the DynaCellect system without Cellmation, as determined via flow cytometry. By day 2, T cells represented >99% of cells isolated. (LP: leukopak)

**Supplementary figure 2:** T cells isolated (without Cellmation) and cultured for two days were effectively activated and expressed increased levels of CD69, CD25 and HLA-DR.

**TCR Knockout**

**Supplementary figure 3:** T cells that underwent non-viral gene editing without the use of automation software showed effective knockout of TCR at days 5 and 12 respectively after initial T cells isolation.

**Edited**

**Unedited**

**Supplementary figure 4:** Viability of edited and unedited T cells that were manufactured without the automation software solution generally remained high throughout the process.

**Unedited**

**Edited**

**Supplementary figure 5:** Both edited cells and unedited T cells expanded efficiently.

**Unedited**

**Edited**

**Supplementary figure 6:** CAR T cells generated using the unautomated method were efficient in killing target NALM6 cells.

* Results are representative of three independent donors

*Red, blue and black represent Donor 1, Donor 2 and Donor 3 respectively
